# Evaluation of the Utility of Capsular Stabilization Devices in a Zonular Fiber Defect Model with the Slit Side View System

**DOI:** 10.1155/2020/5921965

**Published:** 2020-08-08

**Authors:** Tomoyuki Kunishige, Hisaharu Suzuki, Yuji Nakano, Tsutomu Igarashi, Hiroshi Takahashi

**Affiliations:** Department of Ophthalmology, Nippon Medical School, 1-1-5 Sendagi, Bunkyo-ku, Tokyo 113-8603, Japan

## Abstract

Capsular stabilization devices were evaluated in a zonular fiber defect model using the slit side view (SSV) system to confirm their utility for capsular stabilization during phacoemulsification. A zonular fiber defect model was made by cutting Zinn's zonule under observation with a slit lamp microscope in a porcine eye. Phacoemulsification was performed, and the movement of the lens capsule and the depth of the anterior chamber were observed using the SSV in three groups: control group: no surgical instruments used, CE group: a capsule expander was inserted, and CTR group: a capsular tension ring was inserted. In the control group, the equator of the lens was unstable and was easily suctioned to the port of the ultrasound handpiece. The lens capsule was stable in both in the CE and CTR groups. In the CTR group, the equator responsible for the zonular rupture also returned and closed true to its original position. The utility of the capsular stabilization devices in this zonular fiber defect model was confirmed with the SSV system.

## 1. Introduction

Phacoemulsification in cases with Zinn's zonule dialysis, such as pseudoexfoliation or Marfan syndrome, can be troublesome and is associated with an increased risk of intraoperative complications [1–6]. In these cases, the usefulness of devices that expand and support the lens capsule has been reported [7–14], and several types of capsular stabilization devices have been developed. In Japan, two devices are available for Zinn's zonule dialysis cases. One is the capsule expander (CE), which is a T-shaped capsular hook that is used to temporarily hook and support the capsulorhexis edge [10]. The other is the capsular tension ring (CTR), which is inserted in a capsular bag to expand the capsule equator [8, 9]. For safe cataract surgery, understanding the anterior chamber dynamics and the three-dimensional movement of the capsular bag when using these capsular stabilization devices is important.

We previously reported the usefulness of the slit side view (SSV), which is a method to observe the anterior chamber in a cross-sectional view [15]. The SSV allows observation of the movement of ophthalmic viscosurgical devices (OVD) and changes in the anterior chamber depth in accordance with the machine settings during surgery [15]. The purpose of the current study was to evaluate the usefulness of capsular stabilization devices in Zinn's zonular fiber defect model with SSV.

## 2. Materials and Methods

**Figure 1:**
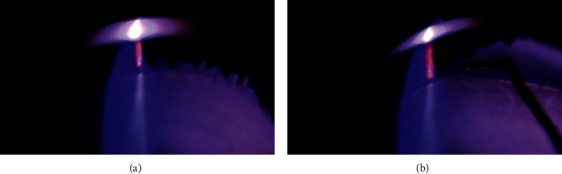
(a) Zinn's zonule was observed with SSV. (b) Zinn's zonule was cut with a phaco chopper.

This study was conducted in accordance with the ARVO Statement for the Use of Animals in Ophthalmic and Vision Research. The extracted porcine eyes used in this experiment were obtained from a local abattoir. Each porcine eye was positioned on an eyeball fixing stand that was attached to a slit lamp microscope (Takagi, Nagano-ken, Japan) [15]. We, then, inserted an OVD via the side port and created a 2.4 mm incised corneal wound. The anterior chamber was filled with the viscoadaptive-type OVD, and then, the OVD was inserted underneath the iris. This allowed observation of the connection of Zinn's zonule, with a visible area ranging from the ciliary process to the lens. After insertion of a phaco chopper hook, Zinn's zonule was cut under direct vision. We were also able to observe the anterior vitreous membrane (Figure 1(a)), which was additionally cut with the hook. We repeated this process approximately 180 degrees to create the zonular fiber defect model (Figure 1(b)). Then, we performed continuous curvilinear capsulorhexis in all eyes. Phacoemulsification was performed with three different settings. In the control, no capsular stabilization devices were used. In the CE group, one CE (Handaya, Tokyo, Japan) was inserted from the opposite side of the main port. In the CTR group, a CTR (Hoya, Tokyo) was inserted from the main port. Phacoemulsification was performed using 20% power of the longitudinal vibration utilizing a 30 degree Signature Laminar® 20-gauge ultrasound (US) tip (AMO Japan K.K., Tokyo, Japan). During the procedures, the movement of the lens capsule and changes in the depth of the anterior chamber were observed with SSV using the slit lamp microscope 700GL (Takagi, Nagano-ken, Japan). Each group included three porcine eyes.

## 3. Results

**Figure 2:**
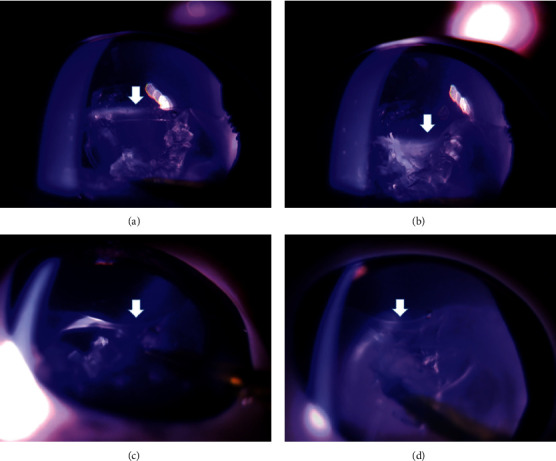
(a) Before phacoemulsification, the equator of the lens was in the correct position (arrow). (b)–(d) During phacoemulsification without any support in the control group, the equator of the lens was unstable (arrow) (*n* = 3).

**Figure 3:**
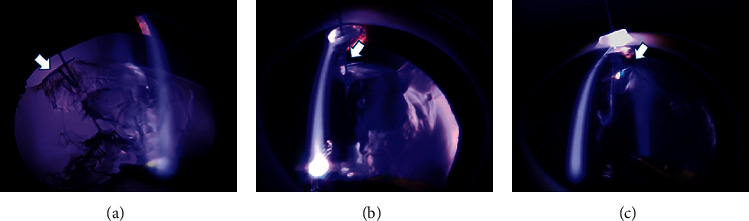
One CE was inserted from the opposite side of the main port (arrow). In the CE group, although the lens equator was stretched, the position of the capsular bag was shifted upward (arrow, a–c, *n* = 3).

In the control group (*n* = 3), phacoemulsification was performed without any support. The equator of the lens was unstable and was easily suctioned into the port of the US hand piece (Figure 2, video S1). In the CE group (*n* = 3), a CE was used to support the lens capsule through the corneal side port. This was effective for stabilizing the capsule with broad zonular fiber defects. Less suction of the equator of the lens was observed. However, because the lens capsule was suspended only in the upper part, the equator of the lens was not in its original position (Figure 3, video S2). In the CTR group (*n* = 3), insertion of a CTR allowed expansion of the lens capsule and supported the remaining Zinn's zonules. The equator of the capsule was maintained close to its original position (Figure video S3).

## 4. Discussion

In this study, we filled the anterior chamber with a viscoadaptive-type OVD, which was also inserted underneath the iris. With SSV, we were able to observe Zinn's zonules and the lens equator that normally cannot be observed from the front view. In the control group, the equator and the posterior capsule of the lens were unstable and easily suctioned into the port, suggesting that in cases of Zinn's zonule dialysis, the lens capsule needs to be extended to avoid the risk of posterior capsule damage. The CE is a device developed to support the lens capsule in Zinn's zonule dialysis during cataract surgery [10]. This flexible device uses a silicone rubber ring for fixation, with its end bifurcating to form a T-shaped footpad [10]. In the CE group, one CE was inserted from the opposite side of the main port. As this device supported the lens capsule through the corneal side port, it was effective for safe aspiration and removal of the lens without aspiration of the capsular bag. However, we observed an upward shift of the position of the capsular bag so that it was not fixed in its original position in the lens equator. In our model, approximately 180 degrees of Zinn's zonule dialysis were supported by one CE, suggesting that even in cases in which the range of the zonule rupture is large, safe phacoemulsification may be possible by increasing the number of CEs. The CTR was invented as an effective ring for maintaining the shape of the crystalline lens in cases with weak Zinn's zonules [9, 11, 16]. In the current study, we confirmed that Zinn's zonule dialysis was stretched by the CTR, which led to stable fixation of the lens equator. In our 180-degree Zinn's zonule dialysis model, when the residual Zinn's zonules were normal, phacoemulsification could be performed without any problems with the CTR.

Yaguchi et al. demonstrated that use of the experimental porcine eye model with zonular dehiscence allows observation of the entire configuration of the lens capsule, and they also demonstrated differences in the efficacy of capsular bag retention with a CTR and CE [17]. In our current study, we demonstrated that with SSV, we were able to observe the intraoperative lens, the anterior chamber dynamics, and the three-dimensional movement of the capsular bag in the zonular fiber defect model. The usefulness of the CE and CTR was confirmed.

## 5. Conclusions

Using SSV, dynamics of the change in the anterior chamber depth and the capsular bag were examined, and the usefulness of the CE and CTR in the zonular fiber defect was confirmed.

## Figures and Tables

**Figure 4:**
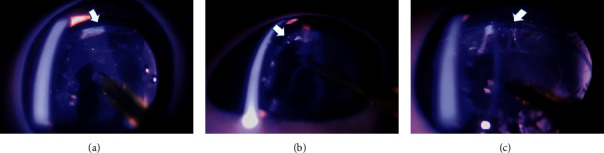
A CTR was inserted from the main port (arrow). The CTR was able to expand the lens and capsule and, thus, support the remaining Zinn's zonules in the original position in the CTR group (arrow, a–c, *n* = 3).

## Data Availability

The data used to support the findings of this study are included within the article.

## Supplementary Materials

Supplementary Materials Video legends: video S1: in the control group, phacoemulsification was performed without any support. The equator of the lens was unstable and was easily suctioned into the port of the US hand piece. Video S2: in the CE group, phacoemulsification was performed with one CE. Although the CE supported the lens capsule through the corneal side port during phacoemulsification, the position of the capsular bag was shifted upward. Video S3: in the CTR group, phacoemulsification was performed with a CTR. SSV demonstrated that the CTR stretched the lens capsule and supported the remaining Zinn's zonules in the original position.
